# Ferulic acid content variation from wheat to bread

**DOI:** 10.1002/fsn3.2171

**Published:** 2021-03-26

**Authors:** Sonia Boudaoud, Delphine Sicard, Lucas Suc, Geneviève Conéjéro, Diego Segond, Chahinez Aouf

**Affiliations:** ^1^ UMR 1083SPO Univ Montpellier INRAE Institut Agro Montpellier France; ^2^ UMR B&PMP INRAE CNRS Univ Montpellier Institut Agro Montpellier France; ^3^ UMR 1208 IATE Univ Montpellier INRAE Institut Agro Montpellier France

**Keywords:** fermentation, ferulic acid, terroir, varieties, Wheat bran

## Abstract

The health‐promoting effects of whole‐grain consumption have been attributed in a large part to the phytochemical profile of the wheat grain, and particularly to the bioactive molecules present in bran. This study shed light on the impact of human practices, especially harvesting sites (terroirs) and wheat species and varieties, as well as bread‐making conditions on the variation of the antioxidant and antimicrobial ferulic acid (FA) content. FA concentration in the bran of wheat species (durum and bread wheat) and varieties (Chevalier, Renan, Redon, Saint Priest le vernois rouge, Bladette de Provence, Pireneo, Rouge de Bordeaux, LA1823, Claudio et Bidi17) harvested in five sites in France on 2015 and 2017, has been evaluated. Statistical analysis showed significant differences in FA content for wheat varieties and terroirs. During bread making, baking and type of leaven impacted the FA content of dough and bread. The differences were not due to the type of fermentation (sourdough/commercial yeast) but rather to the diversity of fermenting microbial strains and flour used for backslopping.

## Graphical abstract

Terroir, wheat varieties as well as fermentation conditions significantly impact the Ferulic acid content in wheat and bread.

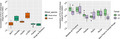

## 1 INTRODUCTION

Wheat is the most important food grain source for humans with an estimated global production of around 747.8 million tons and a consumption that reached 753.9 million tons for the year 2018/2019 (United States Department of Agriculture). This increasing consumption is partly correlated with the nutritional value of the wheat grain which mainly contains carbohydrates (65%–75%), proteins (7%–12%), and lipids (2%–6%) (Pomeranz, 1988). The wheat grain is composed of different parts, of which the bran accounts for 14%–19% of its weight (Javed et al., 2012). Studies on the effect of wheat, oat, and corn brans on human organism showed that wheat bran contains most of the micronutrients and bioactive compounds of the grain (Hemery et al., 2007), and therefore, it might contribute to reduce certain diseases of the digestive system. Wheat bran comprises the outer tissues of the wheat kernel and includes histologically and chemically distinct layers and tissues, whose aleurone layer accounts for 50% of all bran tissue. Aleurone cell walls contain 29% B‐glucans and 65% linear arabinoxylans (Javed et al., 2012; Shewry & Hey, 2015). They are a source of vitamins and bioactive compounds such as phenolic compounds (bound phenolic compounds: 4.73–2020 µg/g) (Onipe et al., 2015). Phenolic compounds of wheat bran mainly belong to phenolic acids group with ferulic acid (4‐hydroxy‐3‐methoxy cinnamic acid) as major component (it represents up to 90% of total phenolic acids) (Adom et al., 2003). Ferulic acid (FA) mainly occurs in *trans* configuration and is esterified to arabinose (linked in the *O‐5* position), stanols, sterols, and glucose (Naczk & Shahidi, 2006). It is also able to be associated to lignin via ether and/or ester linkages. Generally, wheat kernels contain 0.64–1.27 mg/g of FA (Adom & Liu, 2002; Nishizawa et al., 1998).

A number of studies have reported the strong antioxidant activity of FA. DPPH test of different phenolic acids showed that FA has a lower EC50 value (0.0927 µmol/assay) than other counterparts such as vanillic or p‐coumaric acids with EC50 values of 14.34 and 66.29 µmol/assay, respectively (Karamac et al., 2005). FA is also known for its antibacterial and anti‐inflammatory properties (Park et al., 2019). All these properties may be correlated to the established health benefits that may result from the consumption of wheat bran and/or whole wheat grains. Thanks to its antioxidant activity, FA (alone or in synergy with other bioactive compounds) acts as a free radicals scavenger, inhibiting thereby some cardiovascular and coronary heart diseases, along with diabetes (Jones, 2006).

Nutritional and health benefits provided by wheat have driven researches toward assessing the wheat growing factors that influence the content of bioactive components, especially phenolic compounds. Several studies have reported the effect of wheat varieties and species on the phenolic composition. One broad study analyzed the phenolic content of 170 genotypes representing diverse wheat species (bread winter wheat, spring wheat, spelt, durum wheat, cultivated emmer, and einkorn) and showed that the highest FA content was found in winter wheat and its variation depended on varieties (Li et al., 2008). Significant differences between durum and bread wheat varieties were also reported in Italy and USA (Adom et al., 2003; Bordoni et al., 2017). The effect of climate, year of cultivation, and agronomic practices on the FA content has also been previously analyzed (Li et al., 2008). For instance, Shewry and Hey (2015) studied the effect of genotype and environmental variation, including whether conditions, altitude, soil type, temperatures on the phenolic composition of winter and spring wheat. Gasztonyi et al. (2011) evaluated the varietal and agricultural factors, like the use of fungicide, on the phenolic content of different wheat varieties over two years. Heimler et al. (2010) analyzed the polyphenol content of ancient and modern varieties of durum and soft wheat sampled over 2 years. Overall, climate factors and years of cultivation appeared as the main factors that cause variation in polyphenol (Gasztonyi et al., 2011; Pu et al., 2019; Shamloo et al., 2017). However, to our knowledge, no studies have explored the terroir effect. Terroir can be defined as an ecosystem, in a given place, including many factors such as climatic conditions, cultivar rootstock, geography, and topography, as well as soil characteristics like mineral nutrition and water supply. It is also a key index to guide consumers in their search of quality.

Growing wheat with optimal nutritional qualities is useless if the bread we find on our tables, which is the main product of wheat processing is devoid of nutrients. One might wonder whether the different stages of bread‐making influence the evolution of the phenolic compounds present in semi‐wholemeal and wholemeal flours. Angelino et al. (2017) published a recent review summarizing the different operations carried out during bread‐making process that might increase the fraction of phenolic compounds. Fermentation and baking have been reported as the two main factors contributing to the increase in the fraction of FA in bread. Several studies showed that adding yeasts or lactic bacteria to flour or bran have the potential to release insoluble bound FA (Katina, Laitila, et al., 2007; Zhang et al., 2014). In the case of lactic acid bacteria (LAB), the hydrolysis reactions result mainly from the increase in the acidity of the medium. (Hansen et al., 2002; Konopka et al., 2014). During baking, high temperatures may induce structural modifications in plant cell walls, provoking as consequence a rupture of some bounds and the release of some compounds such as FA (Chandrasekara & Shahidi, 2011).

In summary, significant work has been done on the impact of wheat growing conditions on the content of bioactive compounds such as FA. However, no studies have highlighted the influence of terroir on FA content. On the other hand, the analysis of the effect of fermentation in a regular artisanal bread‐making process on FA content was, so far, not reported.

In this work, a set of organic French wheat grains coming from different terroirs, including durum and bread wheat species and belonging to modern and landrace varieties, were studied to compare the FA content of their bran. Then, one modern variety (Renan) has been milled into semi‐Wholemeal flour T80, and the impact of fermentation conditions FA content of dough and bread was assessed. Three different types of fermentation representing different bread‐making practices were compared: fermentation by commercial yeast, fermentation by sourdough, and fermentation by a mix of commercial yeast and sourdough. Finally, the baking effect on bread FA content was evaluated.

## 2 MATERIALS AND METHODS

### 2.1 Wheat samples

Two sets of organic wheat grains kindly provided by Dominique Desclaux (INRAe Domaine de Melgueil, Mauguio) were used. The first one was composed of three varieties of durum wheat (LA1823, Claudio and Bidi17) and three varieties of bread wheat (Chevalier, Pireneo, Rouge de Bordeaux) grown in Mauguio in 2017. Mauguio is a small town located in Hérault in the south of France. In 2017, the average temperature in July and August was 30°C, and the minimum temperature in January was 0°C, with an average annual rainfall of 321 mm (Météo France). The second set contained only grains of bread winter wheat. They were obtained from three modern varieties (Chevalier, Renan, Pireneo) and three landraces (Redon, Bladette de Provence, Saint priest le Vernois Rouge) grown in France in 2015, at four locations: le Rheu (LA) and Chavagne (GS) in Bretagne region; along with Pont de l’Arche (FM) and Le Puits (LM) in Pays de la Loire region. These two regions located in northwestern France, are neighboring regions characterized by a rather wet climate. In 2015, the average rainfall was almost of 647 mm in Bretagne, and 611 mm in Pays de la Loire (Météo France). The maximum recorded temperatures were between 25°C and 27°C in June, and the minimum temperatures of −8°C were recorded in January.

All varieties were grown on terroir GS (Chavagne, Bretagne) and terroir LA (Le Rheu, Bretagne). The modern varieties were also grown on terroir FM (Pont de l’Arche, Pays de la Loire). The landraces were also grown on terroir LM (Le puits, Pays de la Loire). The grains were all kindly provided by Veronique Chable (INRAe Centre Bretagne‐Normandie, UMR BAGAP, Le Rheu, France) and stored at −20°C (Table 1).

**TABLE 1:** Bread wheat varieties harvested on 2015 at four French terroirs LA, GS, FM and LM

Terroir	Variety^a^	
LA: Le Rheu, Bretagne	Landraces	Redon
		Bladette de Provence
		Saint Priest le Vernois Rouge
	Modern	Chevalier
		Renan
		Pireneo
GS: Chavagne, Bretagne	Landraces	Redon
		Bladette de Provence
		Saint Priest le Vernois Rouge
	Modern	Chevalier
		Renan
		Pireneo
FM: Pont de l’Arche, Pays de la Loire	Landraces	Redon
		Bladette de Provence
		Saint Priest le Vernois Rouge
LM: Le puits, Pays de la Loire	Modern	Chevalier
		Renan
		Pireneo

^a^ To perform this study, three seed lots were randomly collected from each variety (biological triplicate).

### 2.2 Wheat grains milling and bran recovery

Wheat grains, with an average weight of 4.74 ± 0.36 g were tempered by moistening at 20% w/v in a rotating system, during 3 hr, and were left at room temperature for 24 hr. Then, the humidified grains were ground in mortar grinder FRITSCH PULVERISETTE2, operating at a speed of 70 to 80 rpm, with 12.5 daN (downforce) on the superior roller and 0 ring on the lateral screw. Ground wheat was sieved through two sieves with 1 mm and 0.5 mm size screens, respectively. Three fractions were obtained, a coarse fraction corresponding to the bran still containing a large part of flour (WBF1: 3.7 ± 0.33 g), an intermediate fraction (IF: 0.45 ± 0.17 g), and a flour fraction (FF1: 0.5 ± 0.18 g). To isolate bran, fraction WBF1 underwent, a second grinding under softer conditions (7.5 daN on the superior roller, 2 ring on the lateral screw). Coarse fraction (WBF2: 1.54 ± 0.16 g), corresponding to the bran, was obtained after sieving through 1 mm size screen sieve (see supporting data for material balance, Table S1). To remove the remaining flour, fraction WBF2 was washed with water (1 g of WBF2 for 100 ml of water) at 50°C, under stirring, for 15 min. After filtration (woven mesh filters, NITEX, SEFAR, with mesh openings of 500 µm), the solid part was washed twice with room temperature water. The washing operation was repeated 3 times. Washed WBF2 was dried overnight at 60°C to obtain the dry bran WBF2‐D.

### 2.3 Dough and bread preparation

Dough and bread samples were prepared using semi‐wholemeal flour (T80) made from Renan wheat variety harvested in 2019. They were prepared in bakery “Le pain Levain” (Azillanet, France) in January 21st, 2020, either by adding commercial yeast, sourdoughs, or a mix of yeast and sourdoughs. Commercial yeast starters were Hirondelle (Lesaffre), Bioreal or Instant (Lesaffre). The three sourdoughs (“CRA”, “EDI”, “STE”,) were chosen for their diversity in yeast species. Sourdough “CRA” contains *Kazachstania bulderi* as dominant yeast species and *L.sanfranciscensis* as dominant LAB species. Sourdough “EDI” contains *Saccharomyces cerevisiae* and *Kazachstania unispora* as codominant yeast species as well as *L. sanfranciscensis* as dominant LAB species. Sourdough “STE” contains *Saccharomyces cerevisiae* and *Torulaspora delbrueckii* as codominant yeast species as well as *L. sanfranciscensis* as dominant LAB species. The yeast/sourdough fermentation was made by mixing the dough made with sourdough (“STE” or “CRA”) and the one made with the commercial yeast Hirondelle (Hirondelle × “STE” sourdough and Hirondelle × “CRA” sourdough).

Doughs were left over for a first fermentation during a time ranging from 1 hr20 to 1 hr55. Then, they were divided by 500 g, shaped and left for a second fermentation that lasted 3 hr35. Dough loafs were then baked in a wood oven at 250°C.

Quantities of ingredients and the conditions of the different steps of bread making are listed in Table S2.

### 2.4 Preparation of sourdough, dough, and bread samples for solvent extraction

Sourdoughs (“CRA”, “STE”, and “EDI”) and doughs were freeze‐dried for 47 hr in a freeze dryer (EDWARDS) to obtain water‐free samples with average weight of 4.73 ± 1.04 g.

Three slices were recovered from each loaf, and two types of samples were prepared from each slice. One was composed of crumb taken in the center of the slide (inner bread samples) and one containing crumb and crust was taken on the border of the slide (outer bread samples). To facilitate the grinding, the samples were dried overnight at 60°C. The average weight of the dried inner samples was 2.6 ± 0.45 g and that of dried outer samples was 3.34 ± 0.77 g.

Biological triplicates were prepared from sourdoughs, doughs, inner, and outer bread samples; then, they were ground on a mortar grinder FRITSCH PULVERISETTE2 with 12.5 daN on the superior roller and 0 rings on the lateral screw, during 2 min for bread samples and 1 min for sourdoughs and doughs.

### 2.5 Extraction of free and hydrolysable FA from wheat bran, sourdough, dough, and bread samples

Bran powder (50 mg), previously ground into fine particles was introduced in a 10 ml Pyrex tube, equipped with a magnetic stirring bar and a screw lid. Then, 1 ml of the extraction solution (2 M NaOH in water/Ethanol 50:50 v/v) was added. The mixture was heated at 80°C for 1 hr under stirring. After cooling down and centrifuging the mixture, 100 µl of the supernatant was collected and neutralized with the same volume of 2 M HCl aqueous solution. To remove the resulting salts, the solution was filtered through Agilent pp 0.2 µm filter.

The extraction of FA from sourdough, dough, and bread samples was carried out in the same manner, by using a solution of 3 M NaOH in water / Ethanol 50:50 v/v, as extraction solution and by heating the samples at 60°C during 4 hr in a dry heating bath, without stirring.

### 2.6 FA quantification from bran, sourdough, dough, and bread samples

The neutralized extraction solutions were injected in Acquity UPLC (Waters, Milford, MA) liquid chromatography system, equipped with a photodiode array detector (DAD). The Waters column was 100 mm × 2.1 mm, HSS T3, particles size was 1.8 µm. The solvent used were A (99% H_2_O and 1%HCOOH v/v) and B (100% CH_3_CN) with a flow rate of 0.55 ml/min. The gradient conditions were as follows: from 0 to 4 min, 99% to 70% A; from 4 min to 7 min, 70% to 20% A; from 7 min to 8 min, 20% A; from 8 min to 9 min, 20% to 99% A. The injection volume was 2 µl and DAD was set at 280 nm (*λ*_max_ of phenolic compounds).

It is well known that the area of a spectral peak is proportional to the amount of the substance that reaches the detector in LC instrument. A response factor was obtained experimentally through calibration curve of commercial *trans*‐FA, obtained by injecting known concentrations ranging from 1 mg/L to 50 mg/L. The mass relative response factor (MRRFx) of FA equals the area of the spectral peak (mAu) divided by its mass concentration. Thus, for the quantitation of FA in the extracts, its mass concentration (Cx) can be computed as: Cx = Ax/MRRFx, where Ax is the peak area of FA.

### 2.7 Statistical analyses

The bran yields as well as FA content in bran, sourdough, dough, and bread samples were analyzed using linear model with the statistical program R version 3.4.4.

#### 2.7.1 Species and variety effect on bran yield and FA content

Data on the first set of grains, composed of durum and bread wheat collected in 2017, was used to analyze the effect of wheat species and varieties using the following linear model:

$$Y_{\mathrm{ijk}}=\mu +{\text{species}}_{i}+{\text{variety}}_{j}{\left(\text{species}\right)}_{i}+\varepsilon_{\mathrm{ijk}},$$

where *Y* is the bran yield or the FA bran content, *µ* is a constant, *species* is the wheat species effect (*i* = 1,2), variety (species) is the wheat variety effect within each species (*j* = 1,2,3), and ε is the experimental error, estimated by the bran yield variation over seed lots of the same variety.

The FA bran content was measured three times independently on three seed lots of each variety. There was no effect of seed lot. The model including the random seed lot effect was less likely than the one without (BIC = −16.04/BIC = −14.34).

#### 2.7.2 Variety and terroir effect on bran yield and FA content

Data on the second set of grains, composed of bread wheat collected in 2015 from different terroirs, were analyzed to test the effect of bread wheat variety and terroir on the bran yield and the FA content, using the following linear model:

$$Y_{\mathrm{ijk}}=\mu +{\text{variety}}_{i}+{\text{terroir}}_{j}+\text{variety}\times {\text{terroir}}_{\mathrm{ij}}+\varepsilon_{\mathrm{ijk}},$$

where *Y* is the quantitative variable (either the bran yield or the FA bran content), µ is a constant, variety is the variety effect (*i* = 1, 2, 3), terroir is the terroirs effect (*j* = 1, 2), terroir × variety is the interaction effect between varieties and the terroirs and ε is the residuals error.

Again, there was no effect of seed lot. The model including the random seed lot effect was less likely than the one without (BIC = 53.43/BIC = 48.75).

#### 2.7.3 Fermentation effect on sourdoughs, doughs, and breads FA content

The fermentation effect was analyzed using the following linear model:

$$Y_{\mathrm{ijk}}=\mu +\text{Fermentation}\_{\text{type}}_{i}+{\text{Leaven}}_{j}\left(\text{Fermentation}\_{\text{type}}_{i}\right)+\varepsilon_{\mathrm{ijk}},$$

where *Y* is the total FA content in doughs or breads, Fermentation_type is the fermentation type (sourdoughs, yeasts or mix), Leaven (Fermentation type) is the leaven effect within the Fermentation type (*j* = 1, 2, 3), *ε* is the residuals error.

The same model without the fermentation type was used to compare FA content among sourdoughs.

Bran yield or the FA content mean differences between pairs of samples (bran, sourdoughs, doughs, breads) were tested using Tukey HSD method.

## 3 RESULTS AND DISCUSSION

Ferulic acid content in bran of different wheat varieties harvested in different farming sites was evaluated. Then, semi‐wholemeal flour T80 made from modern variety Renan (which is the most used in organic agriculture and bakery in France), was processed into breads through different fermentation practices. The impact of fermentations and baking conditions on the evolution of FA content was assessed.

### 3.1 Wheat bran yield: effect of species, terroir, and variety

**FIGURE 1:**
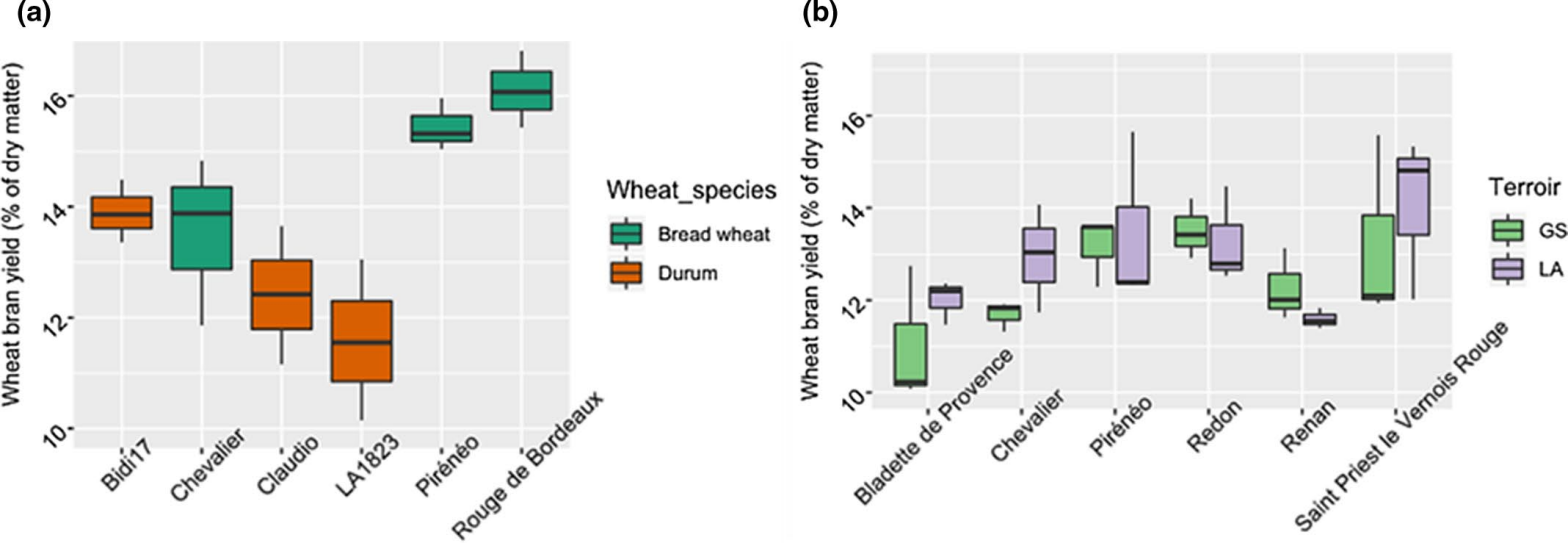
Yield of wheat bran WBF2‐D (% of dry matter). The results are presented with a biological triplicate: (a) On 2017, were harvested the beard wheat varieties: Chevalier, Pireneo, Rouge de Bordeaux and the durum wheat varieties: LA1823, Bidi17, Claudio. (b) The varieties of bread wheat harvested on 2015 are given as follows: Bladette de Provence, Renan, Chevalier, Pireneo, Saint priest le Vernois Rouge, Redon

Tempering is the process of adding water to wheat before milling in order to toughen the bran and soften the endosperm to improve their separation. Many factors are affecting the tempering efficiency such as temperature, moisture, wheat cultivar, and time. Xu et al. (2018) have assessed the effect of moisture level on the softness of the endosperm and concluded that, according to the kernel nature, the wheat grains could be tempered to moisture levels between 13% and 19%. In our case, several tests of tempering/milling have been carried out and the most efficient separation between bran and endosperm was achieved by adding 20% (w/v) of water and tempering at room temperature during 24 hr. After milling, wheat bran WBF2 was washed with warm water four times to reduce as much as possible the starchy part. The bran yields of the wheat varieties studied are displayed on Figure 1.

The wheat varieties generated between 11.52 ± 1.08% to 16.10 ± 0.69% of bran with respect to the grain weight. These results are in agreement with Javed et al. (2012), who reported that approximate bran yield was between 14% and 19% of wheat kernel; and Hemery et al. (2011) study, in which the bran yield was estimated to range between 10% to 15% of wheat grain. The statistical analyses revealed that bran yield does not significally differ between neither wheat species (*F*_1,4_ = 5.39; *p* = .08) in 2017; nor terroirs (*F*_1,24_ = 1.05; *p* = .31) and interaction variety‐terroirs (*F*_5,24_ = 0.58; *p* = .71) in 2015. By contrast, a significant difference was found between wheat varieties harvested in 2017 (*F*_4,12_ = 4.1, *p* = .02) and 2015 (*F*_5,24_ = 3.14; *p* = .02).

For the wheat kernels harvested on 2017 at Mauguio, the highest wheat bran average yield was observed for the bread wheat varieties Rouge de Bordeaux and Pireneo with 16.10 ± 0.69% and 15.44 ± 0.47%, respectively. These two varieties gave a significantly higher bran yield than Claudio and LA1823 (Tukey test, Table S5) with 12.41 ± 1.24% and 11.58 ± 1.45%. The other two varieties (Bidi17 and Chevalier) showed intermediate yields.

Bran yield also significantly changed according to the bread wheat varieties in 2015 although the differences were less marked (*F*_5,24_ = 3.14, *p* = .02). Saint Priest le Vernois Rouge generated the highest amount of bran (13.63 ± 0.73%) and differed from Bladette de Provence (marginally significant Tukey test, Table S6). Interestingly, Pireneo and Chevalier varieties harvested in 2017 at Mauguio presented higher yields than those harvested in 2015 at Bretagne and Pays de la Loire suggesting an effect of the year of culture, environmental, and climate conditions or nature of soil.

In a study conducted by Peyron et al., the mechanical behavior of wheat bran under milling conditions was investigated. It was shown that arabinoxylans which account for 70% of the polysaccharide material in aleurone wall, and their degree of cross‐linking was the main element controlling bran mechanical properties, which in turn control its behavior during milling and then its yield (Peyron et al., 2002). The arabinoxylans content in wheat bran is generally depending on wheat varieties. Indeed, some studies have pointed out the role of breeding in the improvement of arabinoxylans content in wheat varieties (Torok et al., 2019; Tremmel‐Bede et al., 2017). In the other hand, it was demonstrated that there is no differences in mechanical properties between durum wheat and bread wheat bran (Mabille et al., 2001), which is in agreement with our results.

### 3.2 FA content in wheat bran

#### 3.2.1 FA extraction and quantification in wheat bran

Ferulic acid is mostly cross‐linked with lignin and polysaccharides forming lignin/phenolic‐carbohydrates complexes in wheat bran. The chemical extraction of free FA from the vegetable matrix is often performed via alkaline or acid hydrolysis. These methods break the ester bonds between FA and the different molecules of cell walls. However, alkaline hydrolysis is the most effective method. It preserves the structural integrity of FA, while acid hydrolysis causes a cleavage in the glycosidic bonds along with some structural degradations on hydroxycinnamic acids (Aarabi et al., 2016).

**FIGURE 2:**
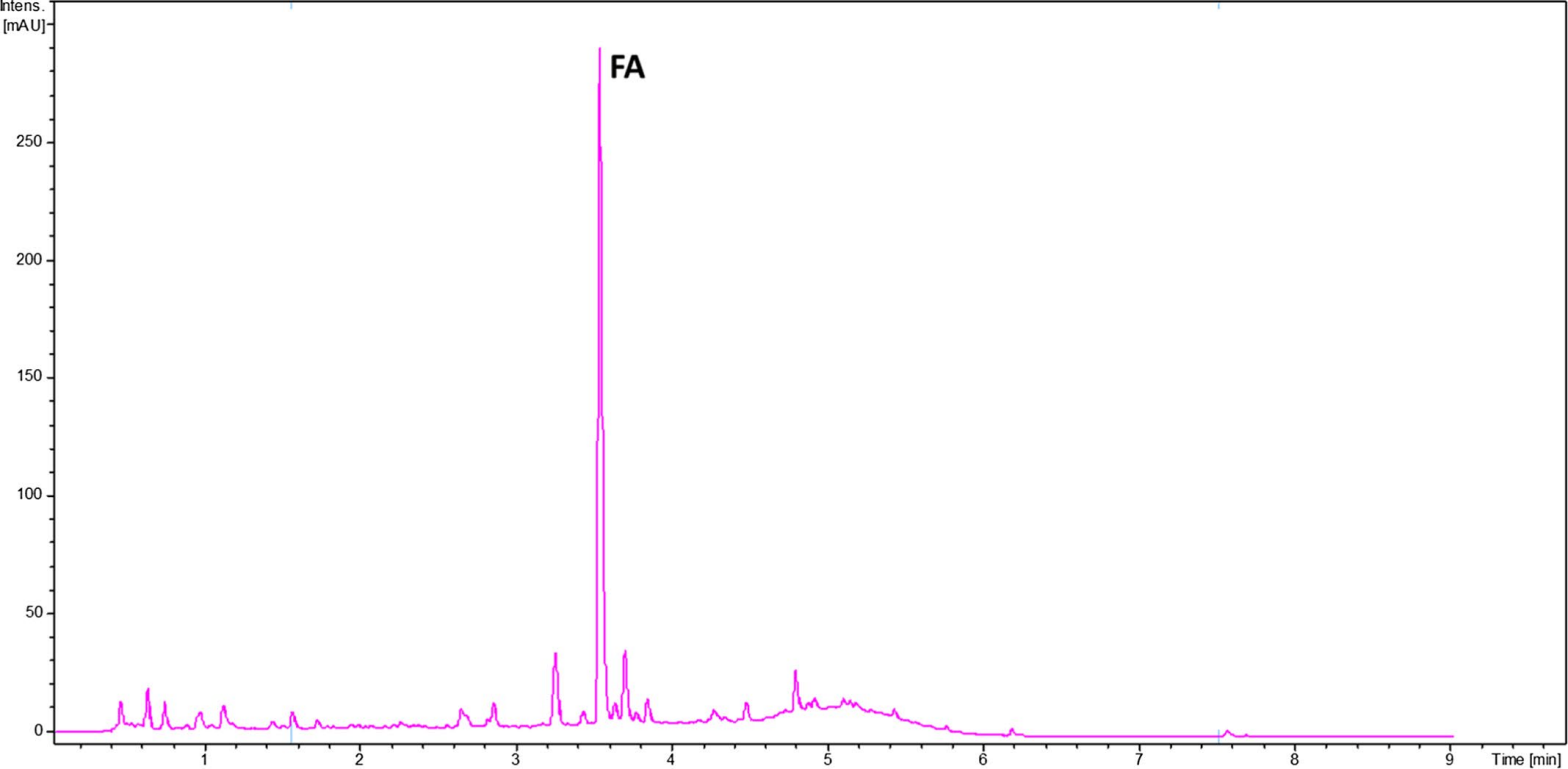
UV chromatogram at 280 nm after alkaline hydrolysis extraction of bran obtained from saint Priest le vernois rouge wheat variety harvested in terroir GS

Ester‐linked FA in the different wheat bran samples was released through alkaline hydrolysis, as shown in Figure 2. The quantification results of FA in wheat brans of the different varieties harvested in 2015 and 2017 are shown on Table 2.

**TABLE 2:** Ferulic acid (FA) contents in wheat bran of varieties harvested in 2015 or 2017. FA content is expressed in mg par g of dry bran

Wheat variety	Terroir/year	Wheat species	FA content in bran (mg/g dry matter)
Bladette de Provence	Pont de l’Arche, Pays de la Loire (FM)/2015	Bread wheat	2.18 ± 0.21
Redon			1.69 ± 0.09
Saint Priest le vernois rouge			1.90 ± 0.17
Bladette de Provence	Chavagne, Bretagne (GS)/2015	Bread wheat	2.51 ± 0.21
Chevalier			2.43 ± 0.21
Pireneo			2.02 ± 0.19
Redon			1.86 ± 0.17
Renan			2.16 ± 0.19
Saint Priest le vernois rouge			2.13 ± 0.31
Bladette de Provence	Le Rheu, Bretagne (LA)/2015	Bread wheat	2.26 ± 0.26
Chevalier			2.37 ± 0.33
Pireneo			1.81 ± 0.18
Redon			1.96 ± 0.33
Renan			2.11 ± 0.26
Saint Priest le vernois rouge			1.81 ± 0.17
Chevalier	Le puits, Pays de la Loire (LM)/2015	Bread wheat	2.04 ± 0.13
Pireneo			2.01 ± 0.10
Renan			2.04 ± 0.25
Chevalier	Mauguio/2017	Bread wheat	1.79 ± 0.18
Pireno			1.38 ± 0.09
Rouge de Bordeaux			1.36 ± 0.08
LA1823	Mauguio/2017	Durum wheat	1.98 ± 0.21
Claudio			1.58 ± 0.08
Bidi17			1.42 ± 0.2

As depicted in Table 2, FA content in the bran of the wheat varieties ranged from 1.36 ± 0.08 to 2.51 ± 0.21 mg/g dm (dry matter). This is in line with other studies that have shown that FA content in wheat bran varies between 0.1 and 14.56 mg/g dm (Liyana‐Pathirana & Shahidi, 2006; Zhou et al., 2004). In comparison, bran of Estonian varieties of spring and winter wheat extracted at room temperature, over 4 hr with 2M NaOH, yielded 0.26 ± 0.05 mg/g dm and 0.53 ± 0.05 mg/g dm, respectively (Vaher et al., 2010). These differences in FA yield could be attributed to the difference in varieties, in geographical localizations of crop, in culture conditions, and in extraction methods.

Although bound forms of FA prevail in the cell walls, the free form could also exist. To check the presence of free FA in our wheat species and varieties, some representative samples of wheat bran of Bladette de Provence from terroir GS, Redon from terroir LA, along with LA1823 and Rouge de Bordeaux harvested in 2017 at Mauguio, were extracted in ethanol/water solvent (50:50 v/v) at 80°C, during 1h. UV quantification revealed that free FA in all these samples was 0.01 ± 0.001 mg/g of dry matter, which represents about 0.5% of total FA (Table S4). Assuming that the samples tested are representative of the other wheat varieties, it appears clearly that the wheat bran samples studied are very poor in free FA.

These results are in agreement with the study reported by Li et al. (2008), which stated that phenolic acids in free form does not exceed 0.5%–1% in cereals. One can infer that the occurrence of FA in wheat bran in linked form might hinder its bioavailability. However, experiments conducted on rats concluded that the bond form of FA is more absorbed in organism than the free one (Rondini et al., 2004). Indeed, the cell wall materials are difficult to digest and may survive upper gastrointestinal digestion, to finally reach the colon. Colonic digestion of such materials by microflora may release the bulk of the bound phytochemicals to exert their health benefits locally and beyond absorption (Liu, 2007). Nevertheless, bioavailability of such compounds varies greatly from bran to bran. In the case of corn bran, FA bound with arabinose and arabinoxylan showed lower bioavailability than free FA (Zhao et al., 2005).

#### 3.2.2 Effect of species, terroir, and variety on the FA content in wheat bran

**FIGURE 3:**
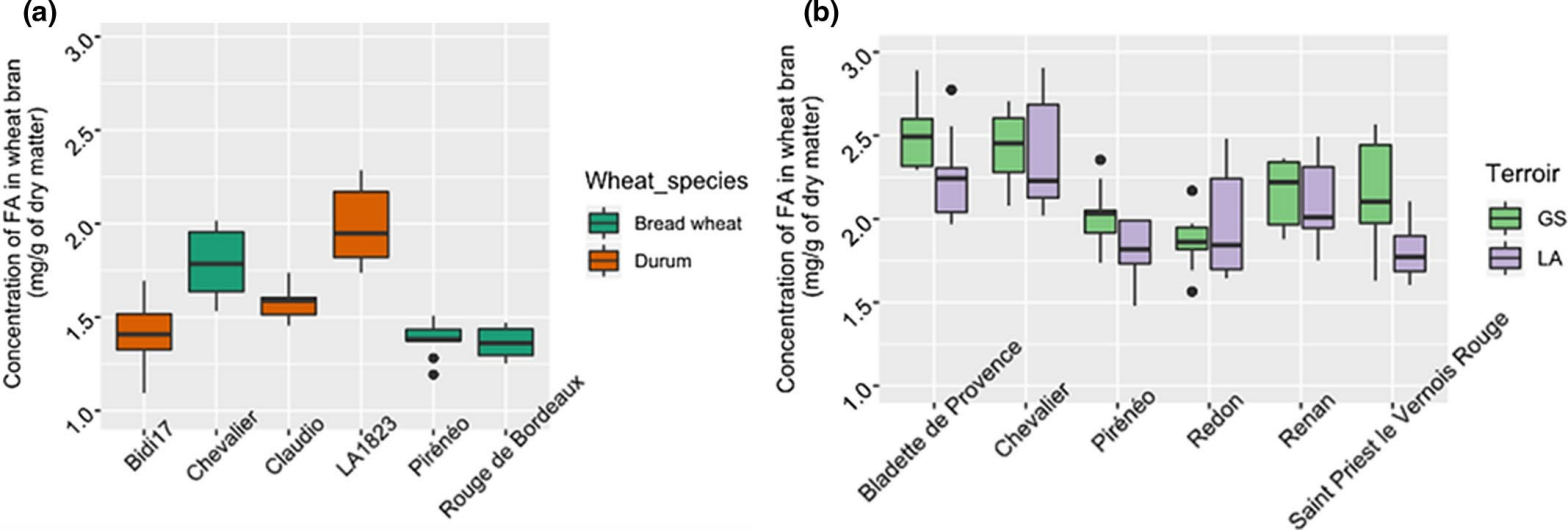
Total FA in the wheat bran of: (a) The durum wheat and the bread wheat varieties harvested on 2017 in Mauguio and (b) the bread wheat varieties, harvested on 2015 on terroirs GS and LA (Bretagne)

As shown in Figure 3, for the wheat varieties harvested in 2017 at Mauguio, we found no significant differences in FA content between wheat species (*F*_1,4_ = 0.49, *p* = .52) and no significant differences between seed lots of the same varieties. By contrast differences appeared among varieties (*F*_4,48_ = 27.08, *p* = 8.72 × 10^–12^). The highest FA content was found in the durum wheat variety LA1823 (1.98 ± 0.21 mg/g) and was significantly different than the FA content in the bread wheat varieties Rouge de Bordeaux, Pireneo and the durum wheat variety Bidi17 which showed the lowest FA contents with concentrations of 1.36 ± 0.08 mg/g, 1.38 ± 0.09, and 1.42 ± 0.2 mg/g, respectively (Tukey test, Table S8). As for bran yield, wheat varieties harvested in 2015 significantly differed in their bran FA content (*F*_5,96_ = 15.65, *p* = 3.0310^–11^). Wheat varieties Chevalier and Bladette de Provence had a significantly higher content than Renan, Saint Priest le Vernois Rouge, Pireneo and Redon (Tukey test, Table S9), with FA concentration of 2.40 ± 0.27, 2.38 ± 0.27, 2.13 ± 0.23, 1.97 ± 0.29, 1.92 ± 0.21, and 1.91 ± 0.26 mg/g, respectively. In addition, a terroir effect was detected (*F*_1,96_ = 7.96, *p* = .0058) with average concentration of 2.19 ± 0.31 mg/g for terroir GS and 2.05 ± 0.33 mg/g for terroir LA. There was no interaction effect between terroir and varieties (*F*_5,96_ = 1.8, *p* = .12).

On the other hand, the wheat variety Chevalier and Pireneo, harvested in Bretagne on 2015, has a higher FA content than that harvested in Mauguio on 2017 (Table 2). This can be attributed to the terroir and/or the year of culture. Indeed, Mauguio region being more warm than Bretagne, it might cause a drop in polyphenols content (Heimler et al., 2010). This result suggests that contrasted terroirs may influence FA content.

The variation of FA content according to the wheat variety has been reported by several studies. In the case of wheat cultivars harvested in USA, the significant difference in FA content was attributed to levels of enzymes involved in phenolic acids metabolism in wheat plant. It was suggested that FA contents were similar during successive phases of grain development, but differences occur in the final concentration, possibly due to FA accumulation and enzyme activities in different cultivars (Adom et al., 2003; Régnier & Macheix, 1996). Other studies explained this variation by the different distribution of phenolic compounds in the bran and endosperm fractions in each variety (Adom et al., 2005).

A number of studies have questioned the effect of modern breeding on the bioactivity and health effect of wheat grains. In other words, do landraces contain more bioactive molecules than modern varieties? Most of these studies (including ours) came to the conclusion that there is no correlation between the type of variety (ancient/modern) and its bioactivity (Bordoni et al., 2017; Shewry & Hey, 2015).

### 3.3 FA in sourdoughs, doughs, and breads

**FIGURE 4:**
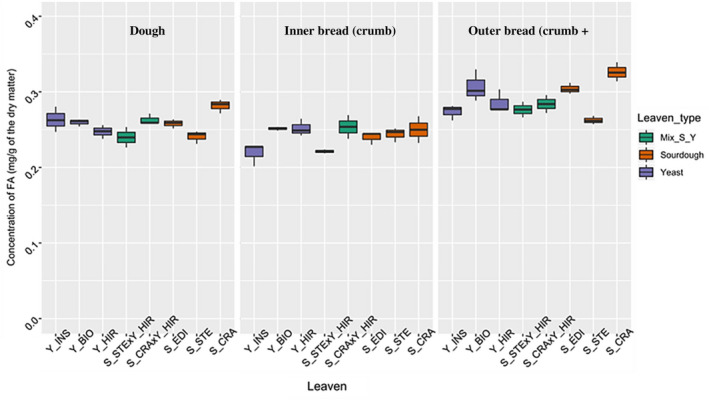
Total FA content in doughs, inner bread samples and outer bread samples, fermented with different leavens. Leavens Y‐CRA, Y‐EDI, Y‐STE represent sourdoughs “CRA”, “EDI”, and “STE”, respectively. Leavens Y‐BIO, Y‐HIR, Y‐INS represent commercial yeasts “Bioreal”, “Hirondelle”, and “Instant”, respectively. Leavens (S‐CRA × Y‐HIR and S‐STE × Y‐HIR) represent mix of sourdough “CRA” and yeast “Hirondelle”, and mix sourdough “STE” and yeast “Hirondelle”, respectively

Total FA content (free and bound) in the different samples of sourdough, dough, and bread are depicted in Table 3 and in Figure 4.

**TABLE 3:** Total ferulic acid (FA) content in sourdough, dough and bread (inner and bread) samples. FA content is expressed in mg/ g of dry matter

Fermentation type	Leaven type	Total FA (mg/g of the dry matter)			
		Sourdough	Dough	Inner bread	Outer bread
Sourdough	CRA	0.52 ± 0.04	0.28 ± 0.009	0.25 ± 0.02	0.33 ± 0.01
	EDI	0.30 ± 0.004	0.26 ± 0.006	0.24 ± 0.008	0.30 ± 0.007
	STE	0.18 ± 0.008	0.24 ± 0.009	0.24 ± 0.009	0.26 ± 0.005
Yeast	BIOREAL	/	0.26 ± 0.005	0.25 ± 0.002	0.30 ± 0.02
	HIR	/	0.25 ± 0.009	0.25 ± 0.01	0.29 ± 0.02
	INSTANT	/	0.26 ± 0.02	0.22 ± 0.01	0.27 ± 0.01
Mix	CRA × HIR	/	0.26 ± 0.007	0.25 ± 0.02	0.28 ± 0.02
	STE × HIR	/	0.24 ± 0.01	0.22 ± 0.004	0.28 ± 0.01

#### 3.3.1 FA content in sourdoughs

Ferulic acid content differed significantly between sourdoughs (*F*_2,6_ = 159.5, *p* = 6.29e−06 ***). It ranged from 0.18 ± 0.008 mg/g dm in sourdough “STE” to 0.52 ± 0.04 mg/g dm in sourdough “CRA”. “EDI” sourdough was an intermediate with a total FA content of 0.3 ± 0.004 mg/g dm. “STE” and “EDI” sourdoughs had significantly different FA content, although they were refreshed with the same flour. This result might be due to the difference in the enzymatic activity of yeast and LAB strains that are able to release bound FA or to transform free FA (Katina, Liukkonen, et al., 2007). Indeed, Ripari et al., have shown that the behavior of sourdough microorganisms toward phenolic compounds during fermentation depends on the species and strains, and on their enzymatic activities. *L. plantarum* strains are able to convert free FA but they do not release bound FA while *L. hammessii* strains released bound FA without degrading it (Ripari et al., 2019).

#### 3.3.2 FA content in doughs before baking

In doughs, the variation of total FA content was between 0.24 ± 0.009 mg/g and 0.28 ± 0.009 mg/g dm. FA content of doughs did not vary according to the fermentation type (*F*_2,5_ = 0.184, *p* = .837) but differed according to the leaven used. Doughs fermented with sourdough “STE”, yeast “HIR”, or a mix of both (sourdough “STE” and yeast “HIR”) showed the lowest FA content (respectively 0.24 ± 0.009, 0.24 ± 0.01, and 0.25 ± 0.009 mg/g dm) while the dough fermented with sourdough “CRA” had the highest FA content (0.28 ± 0.009 mg/g). The differences in acidity between dough fermented with sourdough and dough fermented with yeast alone does not appear to be responsible for the variation in the FA content. Instead, variations in FA content seem to be related to differences between microbial strains. All sourdoughs had *L. sanfranciscensis* as dominant lactic bacteria species and all commercial yeasts were composed of *Saccharomyces cerevisiae* strains. However, sourdough “CRA” used to make the dough with the highest FA content contained *K. bulderi* as dominant yeast species while sourdough “STE” and its mix with “HIR”, used to make the dough with the lowest FA content contained *S. cerevisiae* and *T. debrueckii* as the main yeast strains. Our results suggest that *K. bulderi* may have increased enzyme activity to release bound FA and/or may have a lower AF conversion capacity than *S. cerevisiae* and *T. delbrueckii*.

#### 3.3.3 FA content in breads

It is worth to remind that two type of samples were produced from bread. Inner samples containing only the crumb part of the bread, and outer samples composed of both crumb and crust parts of the bread.

As for doughs, the FA content of breads did not change according to the type of fermentation, that is, sourdough, commercial yeast or mix of the two (*F*_2,5_ = 0.117, *p* = .892 and *F*_2,5_ = 0.28, *p* = .767, for the inner and outer part of the bread, respectively). Instead, a leaven effect was observed (*F*_5,14_ = 4.367, *p* = .013 and *F*_5,14_ = 8.834, *p* < .001) and it was more pronounced for the outer samples of bread. Similarly to doughs, the bread with the highest FA content was made with sourdough “CRA” (0.33 ± 0.01 mg/g) and the one with the lowest FA content was made with sourdough “STE” (0.26 ± 0.005 mg/g).

Finally, baking effect on the bread FA content was tested. A baking effect was observed for both the inner and the outer bread samples (*t* = −3.2646, *p* = 0.01 and *t* = 6.339, *p* < .001, respectively). Compared to the respective doughs, the FA content increased by 0.03 mg/g dm in the outer samples, while it decreased by 0.016 mg/g dm in the inner samples, indicating that the bread crust is richer on FA than the crumb. This is in agreement with Yu et al., results that showed that the total phenolic compounds in breads made from purple wheat wholemeal flour and fermented with commercial yeast, was higher in the crust (0.35 mg of FA equivalent/g) than in the crumb (0.29 mg of FA equivalent/g) (Yu, 2015). Several studies suggested that heat stress can contribute to degrade the cell walls linkages contributing to an increase of free phenolic acids productions (Abdel‐Aal & Rabalski, 2013; Cheng et al., 2006; Gélinas & McKinnon, 2006). As the crust is subjected to higher heat, it is normal for the FA content to be higher in this part.

## 4 CONCLUSION

In an attempt to establish a relationship between wheat nutritional value and human practices from wheat to bread, we studied the effect of wheat varieties, terroir, fermentation, and baking on the FA content. In line with previous studies, we detected significant differences in FA content in bran of wheat varieties, and for the first time, we highlighted the terroir effect on the FA content variation. This FA variation was also affected by the choice of the leaven. Making bread using commercial yeast or sourdough did not appear to have a significant influence. The FA content in doughs and breads was rather impacted by the microbial diversity present in the leaven. Therefore, selecting wheat varieties with good nutritional and health properties remains insufficient if the bread‐making practices are not diversified. Then, exploiting the microbial diversity in sourdoughs is a new avenue for improving bread quality.

## CONFLICT OF INTEREST

There are no conflicts of interest to declare.

## AUTHOR CONTRIBUTIONS

Sonia Boudaoud conducted the experiments related to FA extraction and quantification and contributed to drafting manuscript. Lucas Suc established experimental protocols for wheat grinding and FA characterization. Diego Second contributed to the setting up of experiments for the preparation of sourdough, doughs, and breads. Geneviève Conéjéro contributed to the characterization of FA in wheat bran. Delphine Sicard contributed to the collection of test data, the interpretation of results and the writing of the manuscript. Chahinez Aouf contributed to the design of the study, the interpretation of results and drafting of manuscript.

## Supporting information

Supplementary Material

## Data Availability

The data that supports the findings of this study are available in the Supplementary Material of this article.
