# Supplementary material for: Ferulic acid content variation from wheat to bread
Source: Food Sci Nutr. 2021 Mar 26;9(5):2446–57. doi: 10.1002/fsn3.2171 (PMC8116856; doi:10.1002/fsn3.2171)
Supplement: Supplementary file 1 — Supplementary Material [file FSN3-9-2446-s001.docx]

**Ferulic acid content variation from wheat to bread**

Sonia Boudaoud^1^, Delphine Sicard^1^, Lucas Suc^1^, Geneviève Conéjéro^2^, Diego Segond^1^ and Chahinez Aouf^3^*

**Supplemantary data**

**Table S1**. Weight of all fractions resulting from grinding. The average weights of the fractions are expressed in g of dry matter.

| **Wheat Variety** | **Farming site/year** | **Wheat species** | **Dry grain** | **WBF1** | **IF** | **FF1** | **WBF2** | **FF2*** | **WBF2-D** |
| --- | --- | --- | --- | --- | --- | --- | --- | --- | --- |
| Bladette de Provence | Pont de l’Arche, Pays de la Loire (FM)/2015 | Bread wheat | 4.71± 0.03 | 3.83±0.38 | 0.45±0.1 | 0.39±0.13 | 1.49±0.01 | 2.28±0.28 | 0.54±0.06 |
| Redon |  |  | 4.69±0.03 | 3.99±0.09 | 0.27±0.04 | 0.31±0.04 | 1.51±0.03 | 2.42±0.06 | 0.62±0.03 |
| Saint Priest le vernois rouge |  |  | 4.68±0.05 | 3.57±0.17 | 0.37±0.09 | 0.56±0.1 | 1.29±0.05 | 2.13±0.18 | 0.56±0.006 |
| Bladette de Provence | Chavagne, Bretagne (GS)/2015 | Bread wheat | 4.73±0.03 | 3.63±0.34 | 0.59±0.14 | 0.47±0.18 | 1.45±0.02 | 2.13±0.35 | 0.52±0.07 |
| Chevalier |  |  | 4.68±0.03 | 3.42±0.23 | 0.42±0.02 | 0.72±0.18 | 1.46±0.17 | 1.92±0.08 | 0.55±0.02 |
| Pireneo |  |  | 4.76±0.05 | 3.88±0.33 | 0.44±0.05 | 0.41±0.2 | 1.67±0.07 | 2.16±0.27 | 0.62±0.03 |
| Redon |  |  | 4.76±0.03 | 3.71±0.21 | 0.5±0.13 | 0.51±0.05 | 1.66±0.08 | 1.99±0.14 | 0.64±0.03 |
| Renan |  |  | 4.75±0.04 | 3.84±0.43 | 0.39±0.14 | 0.44±0.2 | 1.56±0.08 | 2.25±0.34 | 0.58±0.04 |
| Saint Priest le vernois rouge |  |  | 4.72±0.03 | 4.07±0.18 | 0.34±0.04 | 0.28±0.08 | 1.59±0.2 | 2.42±0.23 | 0.62±0.1 |
| Bladette de Provence | Le Rheu, Bretagne (LA)/2015 | Bread wheat | 4.61±0.02 | 3.15±0.17 | 0.48±0.07 | 0.86±0.1 | 1.33±0.005 | 1.73±0.17 | 0.55±0.02 |
| Chevalier |  |  | 4.61±0.01 | 3.65±0.08 | 0.33±0.03 | 0.49±0.05 | 1.43±0.06 | 2.08±0.03 | 0.6±0.05 |
| Pireneo |  |  | 4.63±0.06 | 3.58±0.2 | 0.45±0.17 | 0.54±0.06 | 1.49±0.04 | 2.05±0.24 | 0.62±0.08 |
| Redon |  |  | 4.75±0.05 | 3.85±0.35 | 0.43±0.15 | 0.43±0.17 | 1.64±0.12 | 2.16±0.26 | 0.63±0.04 |
| Renan |  |  | 4.69±0.06 | 3.54±0.37 | 0.48±0.12 | 0.6±0.2 | 1.52±0.12 | 1.97±0.25 | 0.54±0001 |
| Saint Priest le vernois rouge |  |  | 4.65±0.02 | 3.84±0.14 | 0.29±0.06 | 0.47±0.1 | 1.64±0.05 | 2.18±0.16 | 0.65±0.08 |
| Chevalier | Le puits, Pays de la Loire (LM)/2015 | Bread wheat | 4.65±0.03 | 3.61±0.09 | 0.5±0.07 | 0.48±0.05 | 1.51±0.04 | 2.08±0.08 | 0.59±0.01 |
| Pireneo |  |  | 4.63±0.02 | 3.6±0.22 | 0.51±0.11 | 0.47±0.07 | 1.42±0.02 | 2.14±0.19 | 0.54±0.02 |
| Renan |  |  | 4.64±0.05 | 3.88±0.31 | 0.39±0.13 | 0.33±0.13 | 1.63±0.16 | 2.21±0.13 | 0.57±0.05 |
| Chevalier | Mauguio /2017 | Bread wheat | 4.74±0.01 | 3.95±0.28 | 0.34±0.17 | 0.43±0.1 | 1.56±0.18 | 2.35±0.25 | 0.64±0.07 |
| Pireno |  |  | 4.71±0.01 | 4.04±0.15 | 0.29±0.01 | 0.41±0.07 | 1.67±0.17 | 2.32±0.3 | 0.73±0.02 |
| Rouge de Bordeaux |  |  | 4.74±0.01 | 4.00±0.08 | 0.37±0.006 | 0.36±0.05 | 1.83±0.22 | 2.14±0.25 | 0.76±0.03 |
| LA1823 | Mauguio /2017 | Durum wheat | 4.75±0.02 | 3.07±0.42 | 0.86±0.3 | 0.82±0.16 | 1.35±0.06 | 1.75±0.41 | 0.55±0.07 |
| Claudio |  |  | 4.75±0.01 | 3.4±0.14 | 0.67±0.1 | 0.69±0.05 | 1.51±0.02 | 1.93±0.17 | 0.59±0.06 |
| Bidi17 |  |  | 4.77±0.02 | 3.66±0.22 | 0.64±0.14 | 0.48±0.09 | 1.78±0.17 | 1.96±0.34 | 0.66±0.02 |

**Table S2.** Quantities of ingredients and conditions of the different steps of bread-making

|  | Bread with commercial yeast | | | Bread with natural sourdough | | | Mix bread | |
| --- | --- | --- | --- | --- | --- | --- | --- | --- |
|  | Hirondelle | Bioreal | Instant | CRA | STE | EDI | HIR x STE | HIR x CRA |
| Flour (Kg) | 21.565 | 21.565 | 21.565 | 21.565 | 21.565 | 21.565 | 21.565 | 21.565 |
| Water (Kg) | 17.37 | 17.37 | 17.37 | 17.37 | 17.37 | 17.37 | 17.37 | 17.37 |
| Salt (g) | 315 | 315 | 315 | 315 | 315 | 315 | 315 | 315 |
| Furnace temperature (°C) | 22 | 22 | 22 | 22 | 22 | 22 | 22 | 22 |
| Flour temperature (°C) | 15 | 15 | 15 | 15 | 15 | 15 | 15 | 15 |
| Water temperature (°C) | 35 | 35 | 35 | 35 | 35 | 35 | 35 | 35 |
| Dough temperature (°C) | 26 | 26 | 26 | 26 | 26 | 26 | 26 | 26 |
| Knead time (min) | 7.55 | 7.55 | 7.55 | 7.55 | 7.55 | 7.55 | 7.55 | 7.55 |
| Kneading speed (unité) | 1 | 1 | 1 | 1 | 1 | 1 | 1 | 1 |
| division into 5 kg of dough | 5 | 5 | 5 | 5 | 5 | 5 | 5 | 5 |
| Sourdough add (Kg) | 0 | 0 | 0 | 0.8 | 0.9 | 0.9 | 1kg of dough + 1kg of dough with “STE” sourdough | 1kg of dough + 1kg of dough with “CRA” sourdough |
| Yeast add (g) | 20 | 30 | 7 | 0 | 0 | 0 | 5g fresh Hirondelle yeast | 5g fresh Hirondelle yeast |
| Sourdough / yeast temperature (°C) | na | 10.5 | 20 | 18.8 | 19 | 20.6 | 21 (dough) | 11 |
| Salt (g) | 0 | 0 | 0 | 15 | 15 | 15 | 0 | 0 |
| Manual incorporation time (min) | 1 | 1 | 1 | 1 | 1 | 1 | 5 | 2 |
| First fermentation time (min) | 113 | 114 | 115 | 113 | 112 | 110 | 80 | 103 |
| Temperature at the end of rounding (°C) | 19.2/ 20.2 | 19.6 | 20.4/19.6/19.6 | 20 | 20.6 | 20.2 | 19/ 19.2 | 19.6/ 19.4 |
| Cooking time (min) | 27 | 27 | 28 | 27 | 28 | 28 | 27 | 29 |
| Oven bread temperature (°C) | 250 | 250 | 250 | 250 | 250 | 250 | 250 | 250 |

**Table S3.** data of wheat bran yield expressed in g (Mass_bran) and in percent of dry wheat grain (Yield_bran). Ferulic acid content in bran of various varieties of durum wheat and bread wheat of different terroirs, expressed by mg / g (FA_bran).

Experimentations were carried out with biological replicate expressed by "Sample" and technical triplicate expressed by "Repetition"

| Year | Wheat_species | Type | Terroir | Variety | TerroirVariety | Sample | Repetition | Mass_bran | Yield_bran | FA_bran |
| --- | --- | --- | --- | --- | --- | --- | --- | --- | --- | --- |
| 2015 | Bread wheat | landraces | FM | Saint Priest le Vernois Rouge | FM_Saint Priest le Vernois Rouge | 1 | 1 | 0.56 | 11.99 | 2.107 |
| 2015 | Bread wheat | landraces | FM | Saint Priest le Vernois Rouge | FM_Saint Priest le Vernois Rouge | 1 | 2 | na | na | 2.121 |
| 2015 | Bread wheat | landraces | FM | Saint Priest le Vernois Rouge | FM_Saint Priest le Vernois Rouge | 1 | 3 | na | na | 2.117 |
| 2015 | Bread wheat | landraces | FM | Saint Priest le Vernois Rouge | FM_Saint Priest le Vernois Rouge | 2 | 1 | 0.56 | 12.07 | 1.71 |
| 2015 | Bread wheat | landraces | FM | Saint Priest le Vernois Rouge | FM_Saint Priest le Vernois Rouge | 2 | 2 | na | na | 1.745 |
| 2015 | Bread wheat | landraces | FM | Saint Priest le Vernois Rouge | FM_Saint Priest le Vernois Rouge | 2 | 3 | na | na | 1.755 |
| 2015 | Bread wheat | landraces | FM | Saint Priest le Vernois Rouge | FM_Saint Priest le Vernois Rouge | 3 | 1 | 0.55 | 11.6 | 1.864 |
| 2015 | Bread wheat | landraces | FM | Saint Priest le Vernois Rouge | FM_Saint Priest le Vernois Rouge | 3 | 2 | na | na | 1.813 |
| 2015 | Bread wheat | landraces | FM | Saint Priest le Vernois Rouge | FM_Saint Priest le Vernois Rouge | 3 | 3 | na | na | 1.879 |
| 2015 | Bread wheat | landraces | GS | Saint Priest le Vernois Rouge | GS_Saint Priest le Vernois Rouge | 1 | 1 | 0.56 | 11.94 | 2.567 |
| 2015 | Bread wheat | landraces | GS | Saint Priest le Vernois Rouge | GS_Saint Priest le Vernois Rouge | 1 | 2 | na | na | 2.443 |
| 2015 | Bread wheat | landraces | GS | Saint Priest le Vernois Rouge | GS_Saint Priest le Vernois Rouge | 1 | 3 | na | na | 2.457 |
| 2015 | Bread wheat | landraces | GS | Saint Priest le Vernois Rouge | GS_Saint Priest le Vernois Rouge | 2 | 1 | 0.74 | 15.58 | 2.028 |
| 2015 | Bread wheat | landraces | GS | Saint Priest le Vernois Rouge | GS_Saint Priest le Vernois Rouge | 2 | 2 | na | na | 1.841 |
| 2015 | Bread wheat | landraces | GS | Saint Priest le Vernois Rouge | GS_Saint Priest le Vernois Rouge | 2 | 3 | na | na | 1.63 |
| 2015 | Bread wheat | landraces | GS | Saint Priest le Vernois Rouge | GS_Saint Priest le Vernois Rouge | 3 | 1 | 0.57 | 12.1 | 1.975 |
| 2015 | Bread wheat | landraces | GS | Saint Priest le Vernois Rouge | GS_Saint Priest le Vernois Rouge | 3 | 2 | na | na | 2.103 |
| 2015 | Bread wheat | landraces | GS | Saint Priest le Vernois Rouge | GS_Saint Priest le Vernois Rouge | 3 | 3 | na | na | 2.128 |
| 2015 | Bread wheat | landraces | LA | Saint Priest le Vernois Rouge | LA_Saint Priest le Vernois Rouge | 1 | 1 | 0.71 | 15.33 | 1.703 |
| 2015 | Bread wheat | landraces | LA | Saint Priest le Vernois Rouge | LA_Saint Priest le Vernois Rouge | 1 | 2 | na | na | 1.66 |
| 2015 | Bread wheat | landraces | LA | Saint Priest le Vernois Rouge | LA_Saint Priest le Vernois Rouge | 1 | 3 | na | na | 1.605 |
| 2015 | Bread wheat | landraces | LA | Saint Priest le Vernois Rouge | LA_Saint Priest le Vernois Rouge | 2 | 1 | 0.69 | 14.81 | 1.772 |
| 2015 | Bread wheat | landraces | LA | Saint Priest le Vernois Rouge | LA_Saint Priest le Vernois Rouge | 2 | 2 | na | na | 1.898 |
| 2015 | Bread wheat | landraces | LA | Saint Priest le Vernois Rouge | LA_Saint Priest le Vernois Rouge | 2 | 3 | na | na | 1.686 |
| 2015 | Bread wheat | landraces | LA | Saint Priest le Vernois Rouge | LA_Saint Priest le Vernois Rouge | 3 | 1 | 0.56 | 12.02 | 2.106 |
| 2015 | Bread wheat | landraces | LA | Saint Priest le Vernois Rouge | LA_Saint Priest le Vernois Rouge | 3 | 2 | na | na | 2.005 |
| 2015 | Bread wheat | landraces | LA | Saint Priest le Vernois Rouge | LA_Saint Priest le Vernois Rouge | 3 | 3 | na | na | 1.86 |
| 2015 | Bread wheat | modern | GS | Chevalier | GS_Chevalier | 1 | 1 | 0.53 | 11.32 | 2.266 |
| 2015 | Bread wheat | modern | GS | Chevalier | GS_Chevalier | 1 | 2 | na | na | 2.453 |
| 2015 | Bread wheat | modern | GS | Chevalier | GS_Chevalier | 1 | 3 | na | na | 2.079 |
| 2015 | Bread wheat | modern | GS | Chevalier | GS_Chevalier | 2 | 1 | 0.55 | 11.83 | 2.69 |
| 2015 | Bread wheat | modern | GS | Chevalier | GS_Chevalier | 2 | 2 | na | na | 2.302 |
| 2015 | Bread wheat | modern | GS | Chevalier | GS_Chevalier | 2 | 3 | na | na | 2.53 |
| 2015 | Bread wheat | modern | GS | Chevalier | GS_Chevalier | 3 | 1 | 0.56 | 11.91 | 2.705 |
| 2015 | Bread wheat | modern | GS | Chevalier | GS_Chevalier | 3 | 2 | na | na | 2.28 |
| 2015 | Bread wheat | modern | GS | Chevalier | GS_Chevalier | 3 | 3 | na | na | 2.604 |
| 2015 | Bread wheat | modern | LA | Chevalier | LA_Chevalier | 1 | 1 | 0.65 | 14.07 | 2.127 |
| 2015 | Bread wheat | modern | LA | Chevalier | LA_Chevalier | 1 | 2 | na | na | 2.021 |
| 2015 | Bread wheat | modern | LA | Chevalier | LA_Chevalier | 1 | 3 | na | na | 2.111 |
| 2015 | Bread wheat | modern | LA | Chevalier | LA_Chevalier | 2 | 1 | 0.54 | 11.74 | 2.905 |
| 2015 | Bread wheat | modern | LA | Chevalier | LA_Chevalier | 2 | 2 | na | na | 2.685 |
| 2015 | Bread wheat | modern | LA | Chevalier | LA_Chevalier | 2 | 3 | na | na | 2.783 |
| 2015 | Bread wheat | modern | LA | Chevalier | LA_Chevalier | 3 | 1 | 0.6 | 13.04 | 2.19 |
| 2015 | Bread wheat | modern | LA | Chevalier | LA_Chevalier | 3 | 2 | na | na | 2.308 |
| 2015 | Bread wheat | modern | LA | Chevalier | LA_Chevalier | 3 | 3 | na | na | 2.228 |
| 2015 | Bread wheat | modern | LM | Chevalier | LM_Chevalier | 1 | 1 | 0.59 | 12.72 | 2.128 |
| 2015 | Bread wheat | modern | LM | Chevalier | LM_Chevalier | 1 | 2 | na | na | 2.059 |
| 2015 | Bread wheat | modern | LM | Chevalier | LM_Chevalier | 1 | 3 | na | na | 2.025 |
| 2015 | Bread wheat | modern | LM | Chevalier | LM_Chevalier | 2 | 1 | 0.6 | 12.88 | 1.913 |
| 2015 | Bread wheat | modern | LM | Chevalier | LM_Chevalier | 2 | 2 | na | na | 1.918 |
| 2015 | Bread wheat | modern | LM | Chevalier | LM_Chevalier | 2 | 3 | na | na | 2.16 |
| 2015 | Bread wheat | modern | LM | Chevalier | LM_Chevalier | 3 | 1 | 0.57 | 12.13 | 2.29 |
| 2015 | Bread wheat | modern | LM | Chevalier | LM_Chevalier | 3 | 2 | na | na | 1.973 |
| 2015 | Bread wheat | modern | LM | Chevalier | LM_Chevalier | 3 | 3 | na | na | 1.937 |
| 2015 | Bread wheat | landraces | FM | Bladette de Provence | FM_Bladette de Provence | 1 | 1 | 0.61 | 12.87 | 2.132 |
| 2015 | Bread wheat | landraces | FM | Bladette de Provence | FM_Bladette de Provence | 1 | 2 | na | na | 2.108 |
| 2015 | Bread wheat | landraces | FM | Bladette de Provence | FM_Bladette de Provence | 1 | 3 | na | na | 2.072 |
| 2015 | Bread wheat | landraces | FM | Bladette de Provence | FM_Bladette de Provence | 2 | 1 | 0.49 | 10.47 | 2.613 |
| 2015 | Bread wheat | landraces | FM | Bladette de Provence | FM_Bladette de Provence | 2 | 2 | na | na | 2.33 |
| 2015 | Bread wheat | landraces | FM | Bladette de Provence | FM_Bladette de Provence | 2 | 3 | na | na | 2.308 |
| 2015 | Bread wheat | landraces | FM | Bladette de Provence | FM_Bladette de Provence | 3 | 1 | 0.53 | 11.28 | 1.906 |
| 2015 | Bread wheat | landraces | FM | Bladette de Provence | FM_Bladette de Provence | 3 | 2 | na | na | 2.053 |
| 2015 | Bread wheat | landraces | FM | Bladette de Provence | FM_Bladette de Provence | 3 | 3 | na | na | 2.125 |
| 2015 | Bread wheat | landraces | GS | Bladette de Provence | GS_Bladette de Provence | 1 | 1 | 0.48 | 10.08 | 2.339 |
| 2015 | Bread wheat | landraces | GS | Bladette de Provence | GS_Bladette de Provence | 1 | 2 | na | na | 2.294 |
| 2015 | Bread wheat | landraces | GS | Bladette de Provence | GS_Bladette de Provence | 1 | 3 | na | na | 2.317 |
| 2015 | Bread wheat | landraces | GS | Bladette de Provence | GS_Bladette de Provence | 2 | 1 | 0.6 | 12.74 | 2.599 |
| 2015 | Bread wheat | landraces | GS | Bladette de Provence | GS_Bladette de Provence | 2 | 2 | na | na | 2.493 |
| 2015 | Bread wheat | landraces | GS | Bladette de Provence | GS_Bladette de Provence | 2 | 3 | na | na | 2.296 |
| 2015 | Bread wheat | landraces | GS | Bladette de Provence | GS_Bladette de Provence | 3 | 1 | 0.48 | 10.23 | 2.589 |
| 2015 | Bread wheat | landraces | GS | Bladette de Provence | GS_Bladette de Provence | 3 | 2 | na | na | 2.891 |
| 2015 | Bread wheat | landraces | GS | Bladette de Provence | GS_Bladette de Provence | 3 | 3 | na | na | 2.739 |
| 2015 | Bread wheat | landraces | LA | Bladette de Provence | LA_Bladette de Provence | 1 | 1 | 0.56 | 12.2 | 2.041 |
| 2015 | Bread wheat | landraces | LA | Bladette de Provence | LA_Bladette de Provence | 1 | 2 | na | na | 1.969 |
| 2015 | Bread wheat | landraces | LA | Bladette de Provence | LA_Bladette de Provence | 1 | 3 | na | na | 2.017 |
| 2015 | Bread wheat | landraces | LA | Bladette de Provence | LA_Bladette de Provence | 2 | 1 | 0.53 | 11.47 | 2.182 |
| 2015 | Bread wheat | landraces | LA | Bladette de Provence | LA_Bladette de Provence | 2 | 2 | na | na | 2.305 |
| 2015 | Bread wheat | landraces | LA | Bladette de Provence | LA_Bladette de Provence | 2 | 3 | na | na | 2.243 |
| 2015 | Bread wheat | landraces | LA | Bladette de Provence | LA_Bladette de Provence | 3 | 1 | 0.57 | 12.36 | 2.774 |
| 2015 | Bread wheat | landraces | LA | Bladette de Provence | LA_Bladette de Provence | 3 | 2 | na | na | 2.556 |
| 2015 | Bread wheat | landraces | LA | Bladette de Provence | LA_Bladette de Provence | 3 | 3 | na | na | 2.245 |
| 2015 | Bread wheat | modern | GS | Pirénéo | GS_Pirénéo | 1 | 1 | 0.59 | 12.29 | 1.966 |
| 2015 | Bread wheat | modern | GS | Pirénéo | GS_Pirénéo | 1 | 2 | na | na | 2.354 |
| 2015 | Bread wheat | modern | GS | Pirénéo | GS_Pirénéo | 1 | 3 | na | na | 2.241 |
| 2015 | Bread wheat | modern | GS | Pirénéo | GS_Pirénéo | 2 | 1 | 0.64 | 13.59 | 1.737 |
| 2015 | Bread wheat | modern | GS | Pirénéo | GS_Pirénéo | 2 | 2 | na | na | 1.917 |
| 2015 | Bread wheat | modern | GS | Pirénéo | GS_Pirénéo | 2 | 3 | na | na | 1.855 |
| 2015 | Bread wheat | modern | GS | Pirénéo | GS_Pirénéo | 3 | 1 | 0.64 | 13.59 | 2.05 |
| 2015 | Bread wheat | modern | GS | Pirénéo | GS_Pirénéo | 3 | 2 | na | na | 2.041 |
| 2015 | Bread wheat | modern | GS | Pirénéo | GS_Pirénéo | 3 | 3 | na | na | 2.033 |
| 2015 | Bread wheat | modern | LA | Pirénéo | LA_Pirénéo | 1 | 1 | 0.72 | 15.65 | 1.479 |
| 2015 | Bread wheat | modern | LA | Pirénéo | LA_Pirénéo | 1 | 2 | na | na | 1.732 |
| 2015 | Bread wheat | modern | LA | Pirénéo | LA_Pirénéo | 1 | 3 | na | na | 1.597 |
| 2015 | Bread wheat | modern | LA | Pirénéo | LA_Pirénéo | 2 | 1 | 0.58 | 12.34 | 1.996 |
| 2015 | Bread wheat | modern | LA | Pirénéo | LA_Pirénéo | 2 | 2 | na | na | 1.802 |
| 2015 | Bread wheat | modern | LA | Pirénéo | LA_Pirénéo | 2 | 3 | na | na | 1.818 |
| 2015 | Bread wheat | modern | LA | Pirénéo | LA_Pirénéo | 3 | 1 | 0.57 | 12.39 | 1.994 |
| 2015 | Bread wheat | modern | LA | Pirénéo | LA_Pirénéo | 3 | 2 | na | na | 1.907 |
| 2015 | Bread wheat | modern | LA | Pirénéo | LA_Pirénéo | 3 | 3 | na | na | 1.991 |
| 2015 | Bread wheat | modern | LM | Pirénéo | LM_Pirénéo | 1 | 1 | 0.55 | 11.9 | 2.08 |
| 2015 | Bread wheat | modern | LM | Pirénéo | LM_Pirénéo | 1 | 2 | na | na | 1.804 |
| 2015 | Bread wheat | modern | LM | Pirénéo | LM_Pirénéo | 1 | 3 | na | na | 1.954 |
| 2015 | Bread wheat | modern | LM | Pirénéo | LM_Pirénéo | 2 | 1 | 0.55 | 11.83 | 2.131 |
| 2015 | Bread wheat | modern | LM | Pirénéo | LM_Pirénéo | 2 | 2 | na | na | 2.046 |
| 2015 | Bread wheat | modern | LM | Pirénéo | LM_Pirénéo | 2 | 3 | na | na | 1.992 |
| 2015 | Bread wheat | modern | LM | Pirénéo | LM_Pirénéo | 3 | 1 | 0.52 | 11.28 | 2.091 |
| 2015 | Bread wheat | modern | LM | Pirénéo | LM_Pirénéo | 3 | 2 | na | na | 1.927 |
| 2015 | Bread wheat | modern | LM | Pirénéo | LM_Pirénéo | 3 | 3 | na | na | 2.057 |
| 2015 | Bread wheat | landraces | FM | Redon | FM_Redon | 1 | 1 | 0.63 | 13.38 | 1.686 |
| 2015 | Bread wheat | landraces | FM | Redon | FM_Redon | 1 | 2 | na | na | 1.513 |
| 2015 | Bread wheat | landraces | FM | Redon | FM_Redon | 1 | 3 | na | na | 1.588 |
| 2015 | Bread wheat | landraces | FM | Redon | FM_Redon | 2 | 1 | 0.58 | 12.37 | 1.754 |
| 2015 | Bread wheat | landraces | FM | Redon | FM_Redon | 2 | 2 | na | na | 1.727 |
| 2015 | Bread wheat | landraces | FM | Redon | FM_Redon | 2 | 3 | na | na | 1.69 |
| 2015 | Bread wheat | landraces | FM | Redon | FM_Redon | 3 | 1 | 0.64 | 13.73 | 1.776 |
| 2015 | Bread wheat | landraces | FM | Redon | FM_Redon | 3 | 2 | na | na | 1.791 |
| 2015 | Bread wheat | landraces | FM | Redon | FM_Redon | 3 | 3 | na | na | 1.669 |
| 2015 | Bread wheat | landraces | GS | Redon | GS_Redon | 1 | 1 | 0.611 | 12.92 | 2.169 |
| 2015 | Bread wheat | landraces | GS | Redon | GS_Redon | 1 | 2 | na | na | 1.972 |
| 2015 | Bread wheat | landraces | GS | Redon | GS_Redon | 1 | 3 | na | na | 1.856 |
| 2015 | Bread wheat | landraces | GS | Redon | GS_Redon | 2 | 1 | 0.64 | 13.42 | 1.565 |
| 2015 | Bread wheat | landraces | GS | Redon | GS_Redon | 2 | 2 | na | na | 1.818 |
| 2015 | Bread wheat | landraces | GS | Redon | GS_Redon | 2 | 3 | na | na | 1.693 |
| 2015 | Bread wheat | landraces | GS | Redon | GS_Redon | 3 | 1 | 0.68 | 14.2 | 1.886 |
| 2015 | Bread wheat | landraces | GS | Redon | GS_Redon | 3 | 2 | na | na | 1.862 |
| 2015 | Bread wheat | landraces | GS | Redon | GS_Redon | 3 | 3 | na | na | 1.949 |
| 2015 | Bread wheat | landraces | LA | Redon | LA_Redon | 1 | 1 | 0.61 | 12.79 | 2.481 |
| 2015 | Bread wheat | landraces | LA | Redon | LA_Redon | 1 | 2 | na | na | 2.427 |
| 2015 | Bread wheat | landraces | LA | Redon | LA_Redon | 1 | 3 | na | na | 2.242 |
| 2015 | Bread wheat | landraces | LA | Redon | LA_Redon | 2 | 1 | 0.6 | 12.53 | 1.847 |
| 2015 | Bread wheat | landraces | LA | Redon | LA_Redon | 2 | 2 | na | na | 1.793 |
| 2015 | Bread wheat | landraces | LA | Redon | LA_Redon | 2 | 3 | na | na | 1.843 |
| 2015 | Bread wheat | landraces | LA | Redon | LA_Redon | 3 | 1 | 0.68 | 14.47 | 1.691 |
| 2015 | Bread wheat | landraces | LA | Redon | LA_Redon | 3 | 2 | na | na | 1.698 |
| 2015 | Bread wheat | landraces | LA | Redon | LA_Redon | 3 | 3 | na | na | 1.645 |
| 2015 | Bread wheat | modern | GS | Renan | GS_Renan | 1 | 1 | 0.55 | 11.63 | 2.127 |
| 2015 | Bread wheat | modern | GS | Renan | GS_Renan | 1 | 2 | na | na | 2.344 |
| 2015 | Bread wheat | modern | GS | Renan | GS_Renan | 1 | 3 | na | na | 2.22 |
| 2015 | Bread wheat | modern | GS | Renan | GS_Renan | 2 | 1 | 0.567 | 12.01 | 2.285 |
| 2015 | Bread wheat | modern | GS | Renan | GS_Renan | 2 | 2 | na | na | 2.362 |
| 2015 | Bread wheat | modern | GS | Renan | GS_Renan | 2 | 3 | na | na | 2.34 |
| 2015 | Bread wheat | modern | GS | Renan | GS_Renan | 3 | 1 | 0.63 | 13.13 | 1.912 |
| 2015 | Bread wheat | modern | GS | Renan | GS_Renan | 3 | 2 | na | na | 1.966 |
| 2015 | Bread wheat | modern | GS | Renan | GS_Renan | 3 | 3 | na | na | 1.879 |
| 2015 | Bread wheat | modern | LA | Renan | LA_Renan | 1 | 1 | 0.55 | 11.83 | 1.937 |
| 2015 | Bread wheat | modern | LA | Renan | LA_Renan | 1 | 2 | na | na | 1.997 |
| 2015 | Bread wheat | modern | LA | Renan | LA_Renan | 1 | 3 | na | na | 1.752 |
| 2015 | Bread wheat | modern | LA | Renan | LA_Renan | 2 | 1 | 0.53 | 11.4 | 2.487 |
| 2015 | Bread wheat | modern | LA | Renan | LA_Renan | 2 | 2 | na | na | 2.494 |
| 2015 | Bread wheat | modern | LA | Renan | LA_Renan | 2 | 3 | na | na | 2.312 |
| 2015 | Bread wheat | modern | LA | Renan | LA_Renan | 3 | 1 | 0.55 | 11.55 | 2.022 |
| 2015 | Bread wheat | modern | LA | Renan | LA_Renan | 3 | 2 | na | na | 1.945 |
| 2015 | Bread wheat | modern | LA | Renan | LA_Renan | 3 | 3 | na | na | 2.01 |
| 2015 | Bread wheat | modern | LM | Renan | LM_Renan | 1 | 1 | 0.61 | 13.26 | 2.138 |
| 2015 | Bread wheat | modern | LM | Renan | LM_Renan | 1 | 2 | na | na | 2.318 |
| 2015 | Bread wheat | modern | LM | Renan | LM_Renan | 1 | 3 | na | na | 1.992 |
| 2015 | Bread wheat | modern | LM | Renan | LM_Renan | 2 | 1 | 0.59 | 12.55 | 1.704 |
| 2015 | Bread wheat | modern | LM | Renan | LM_Renan | 2 | 2 | na | na | 1.749 |
| 2015 | Bread wheat | modern | LM | Renan | LM_Renan | 2 | 3 | na | na | 1.768 |
| 2015 | Bread wheat | modern | LM | Renan | LM_Renan | 3 | 1 | 0.51 | 11.04 | 2.191 |
| 2015 | Bread wheat | modern | LM | Renan | LM_Renan | 3 | 2 | na | na | 2.161 |
| 2015 | Bread wheat | modern | LM | Renan | LM_Renan | 3 | 3 | na | na | 2.358 |
| 2017 | Bread wheat | modern | Maugio | Chevalier | Maugio_Chevalier | 1 | 1 | 0.7 | 14.83 | 1.637 |
| 2017 | Bread wheat | modern | Maugio | Chevalier | Maugio_Chevalier | 1 | 2 | na | na | 1.533 |
| 2017 | Bread wheat | modern | Maugio | Chevalier | Maugio_Chevalier | 1 | 3 | na | na | 1.587 |
| 2017 | Bread wheat | modern | Maugio | Chevalier | Maugio_Chevalier | 2 | 1 | 0.56 | 11.86 | 2.016 |
| 2017 | Bread wheat | modern | Maugio | Chevalier | Maugio_Chevalier | 2 | 2 | na | na | 1.998 |
| 2017 | Bread wheat | modern | Maugio | Chevalier | Maugio_Chevalier | 2 | 3 | na | na | 1.955 |
| 2017 | Bread wheat | modern | Maugio | Chevalier | Maugio_Chevalier | 3 | 1 | 0.65 | 13.88 | 1.785 |
| 2017 | Bread wheat | modern | Maugio | Chevalier | Maugio_Chevalier | 3 | 2 | na | na | 1.712 |
| 2017 | Bread wheat | modern | Maugio | Chevalier | Maugio_Chevalier | 3 | 3 | na | na | 1.88 |
| 2017 | Bread wheat | modern | Maugio | Pirénéo | Maugio_Pirénéo | 1 | 1 | 0.71 | 15.042 | 1.508 |
| 2017 | Bread wheat | modern | Maugio | Pirénéo | Maugio_Pirénéo | 1 | 2 | na | na | 1.38 |
| 2017 | Bread wheat | modern | Maugio | Pirénéo | Maugio_Pirénéo | 1 | 3 | na | na | 1.434 |
| 2017 | Bread wheat | modern | Maugio | Pirénéo | Maugio_Pirénéo | 2 | 1 | 0.72 | 15.32 | 1.42 |
| 2017 | Bread wheat | modern | Maugio | Pirénéo | Maugio_Pirénéo | 2 | 2 | na | na | 1.373 |
| 2017 | Bread wheat | modern | Maugio | Pirénéo | Maugio_Pirénéo | 2 | 3 | na | na | 1.44 |
| 2017 | Bread wheat | modern | Maugio | Pirénéo | Maugio_Pirénéo | 3 | 1 | 0.75 | 15.96 | 1.193 |
| 2017 | Bread wheat | modern | Maugio | Pirénéo | Maugio_Pirénéo | 3 | 2 | na | na | 1.281 |
| 2017 | Bread wheat | modern | Maugio | Pirénéo | Maugio_Pirénéo | 3 | 3 | na | na | 1.382 |
| 2017 | Bread wheat | landraces | Maugio | Rouge de Bordeaux | Maugio_Rouge de Bordeaux | 1 | 1 | 0.79 | 16.81 | 1.361 |
| 2017 | Bread wheat | landraces | Maugio | Rouge de Bordeaux | Maugio_Rouge de Bordeaux | 1 | 2 | na | na | 1.296 |
| 2017 | Bread wheat | landraces | Maugio | Rouge de Bordeaux | Maugio_Rouge de Bordeaux | 1 | 3 | na | na | 1.253 |
| 2017 | Bread wheat | landraces | Maugio | Rouge de Bordeaux | Maugio_Rouge de Bordeaux | 2 | 1 | 0.73 | 15.43 | 1.286 |
| 2017 | Bread wheat | landraces | Maugio | Rouge de Bordeaux | Maugio_Rouge de Bordeaux | 2 | 2 | na | na | 1.396 |
| 2017 | Bread wheat | landraces | Maugio | Rouge de Bordeaux | Maugio_Rouge de Bordeaux | 2 | 3 | na | na | 1.311 |
| 2017 | Bread wheat | landraces | Maugio | Rouge de Bordeaux | Maugio_Rouge de Bordeaux | 3 | 1 | 0.76 | 16.07 | 1.47 |
| 2017 | Bread wheat | landraces | Maugio | Rouge de Bordeaux | Maugio_Rouge de Bordeaux | 3 | 2 | na | na | 1.436 |
| 2017 | Bread wheat | landraces | Maugio | Rouge de Bordeaux | Maugio_Rouge de Bordeaux | 3 | 3 | na | na | 1.47 |
| 2017 | Durum | Unknown | Maugio | LA1823 | Maugio_LA1823 | 1 | 1 | 0.55 | 11.55 | 2.17 |
| 2017 | Durum | Unknown | Maugio | LA1823 | Maugio_LA1823 | 1 | 2 | na | na | 2.28 |
| 2017 | Durum | Unknown | Maugio | LA1823 | Maugio_LA1823 | 1 | 3 | na | na | 2.286 |
| 2017 | Durum | Unknown | Maugio | LA1823 | Maugio_LA1823 | 2 | 1 | 0.48 | 10.15 | 1.948 |
| 2017 | Durum | Unknown | Maugio | LA1823 | Maugio_LA1823 | 2 | 2 | na | na | 1.999 |
| 2017 | Durum | Unknown | Maugio | LA1823 | Maugio_LA1823 | 2 | 3 | na | na | 1.847 |
| 2017 | Durum | Unknown | Maugio | LA1823 | Maugio_LA1823 | 3 | 1 | 0.62 | 13.05 | 1.759 |
| 2017 | Durum | Unknown | Maugio | LA1823 | Maugio_LA1823 | 3 | 2 | na | na | 1.737 |
| 2017 | Durum | Unknown | Maugio | LA1823 | Maugio_LA1823 | 3 | 3 | na | na | 1.82 |
| 2017 | Durum | Unknown | Maugio | Bidi17 | Maugio_Bidi17 | 1 | 1 | 0.69 | 14.49 | 1.662 |
| 2017 | Durum | Unknown | Maugio | Bidi17 | Maugio_Bidi17 | 1 | 2 | na | na | 1.517 |
| 2017 | Durum | Unknown | Maugio | Bidi17 | Maugio_Bidi17 | 1 | 3 | na | na | 1.694 |
| 2017 | Durum | Unknown | Maugio | Bidi17 | Maugio_Bidi17 | 2 | 1 | 0.66 | 13.86 | 1.408 |
| 2017 | Durum | Unknown | Maugio | Bidi17 | Maugio_Bidi17 | 2 | 2 | na | na | 1.399 |
| 2017 | Durum | Unknown | Maugio | Bidi17 | Maugio_Bidi17 | 2 | 3 | na | na | 1.5 |
| 2017 | Durum | Unknown | Maugio | Bidi17 | Maugio_Bidi17 | 3 | 1 | 0.64 | 13.36 | 1.214 |
| 2017 | Durum | Unknown | Maugio | Bidi17 | Maugio_Bidi17 | 3 | 2 | na | na | 1.326 |
| 2017 | Durum | Unknown | Maugio | Bidi17 | Maugio_Bidi17 | 3 | 3 | na | na | 1.095 |
| 2017 | Durum | Unknown | Maugio | Claudio | Maugio_Claudio | 1 | 1 | 0.53 | 11.16 | 1.569 |
| 2017 | Durum | Unknown | Maugio | Claudio | Maugio_Claudio | 1 | 2 | na | na | 1.511 |
| 2017 | Durum | Unknown | Maugio | Claudio | Maugio_Claudio | 1 | 3 | na | na | 1.665 |
| 2017 | Durum | Unknown | Maugio | Claudio | Maugio_Claudio | 2 | 1 | 0.59 | 12.42 | 1.514 |
| 2017 | Durum | Unknown | Maugio | Claudio | Maugio_Claudio | 2 | 2 | na | na | 1.737 |
| 2017 | Durum | Unknown | Maugio | Claudio | Maugio_Claudio | 2 | 3 | na | na | 1.588 |
| 2017 | Durum | Unknown | Maugio | Claudio | Maugio_Claudio | 3 | 1 | 0.65 | 13.65 | 1.455 |
| 2017 | Durum | Unknown | Maugio | Claudio | Maugio_Claudio | 3 | 2 | na | na | 1.604 |
| 2017 | Durum | Unknown | Maugio | Claudio | Maugio_Claudio | 3 | 3 | na | na | 1.593 |

**Table S4.** Concentration of free FA in some representative samples, expressed on mg / g of dry bran.

| Year | Wheat_species | Type | Terroir | Variety | Repetition | Free FA |
| --- | --- | --- | --- | --- | --- | --- |
| 2015 | Bread wheat | landraces | GS | Bladette de Provence | 1 | 0.012 |
| 2015 | Bread wheat | landraces | GS | Bladette de Provence | 2 | 0.010 |
| 2015 | Bread wheat | landraces | GS | Bladette de Provence | 3 | 0.010 |
| 2015 | Bread wheat | landraces | LA | Redon | 1 | 0.013 |
| 2015 | Bread wheat | landraces | LA | Redon | 2 | 0.013 |
| 2015 | Bread wheat | landraces | LA | Redon | 3 | 0.013 |
| 2017 | Bread wheat | landraces | Maugio | Rouge de Bordeaux | 1 | 0.010 |
| 2017 | Bread wheat | landraces | Maugio | Rouge de Bordeaux | 2 | 0.010 |
| 2017 | Bread wheat | landraces | Maugio | Rouge de Bordeaux | 3 | 0.009 |
| 2017 | Durum | Unknown | Maugio | LA1823 | 1 | 0.011 |
| 2017 | Durum | Unknown | Maugio | LA1823 | 2 | 0.010 |
| 2017 | Durum | Unknown | Maugio | LA1823 | 3 | 0.010 |

**Tuckey test results**

**1-Wheat bran yield:**

**Table S5:** Comparison of the wheat bran yield in durum wheat and bread wheat, harvested in 2017

The results are expressed with average percentage of dry matter

G1, G2, express groups of varieties. Varieties with the same letters are not significantly different

| Sample | mean value | G1 | G2 |
| --- | --- | --- | --- |
| Rouge de Bordeaux | 16.10 | a |  |
| Pirénéo | 15.44 | a |  |
| Bidi17 | 13.90 | a | b |
| Chevalier | 13.52 | a | b |
| Claudio | 12.41 |  | b |
| LA1823 | 11.58 |  | b |

**Table S6:** Comparison of the wheat bran yield in the bread wheat varieties harvested in 2015, in terroirs GS and LA.

The results are expressed with average percentage of dry matter.

G1 expresses group of varieties. Varieties with the same letters are not significantly different

| Sample | mean value | G1 |
| --- | --- | --- |
| Saint Priest le Vernois Rouge | 13.63 | a |
| Redon | 13.39 | a |
| Pirénéo | 13.31 | a |
| Chevalier | 12.32 | a |
| Renan | 11.93 | a |
| Bladette de Provence | 11.51 | a |

**Table S7:** Comparison of the wheat bran yield in the bread wheat varieties harvested in 2015, in the four terroirs GS, LA, LM and FM.

The results are expressed with average percentage of dry matter.

G1 expresses group of varieties. Varieties with the same letters are not significantly different

| Sample | mean value | G1 |
| --- | --- | --- |
| LA_Saint Priest le Vernois Rouge | 14.05 | a |
| GS_Redon | 13.51 | a |
| LA_Pirénéo | 13.46 | a |
| LA_Redon | 13.26 | a |
| GS_Saint Priest le Vernois Rouge | 13.21 | a |
| FM_Redon | 13.16 | a |
| GS_Pirénéo | 13.16 | a |
| LA_Chevalier | 12.95 | a |
| LM_Chevalier | 12.58 | a |
| LM_Renan | 12.28 | a |
| GS_Renan | 12.26 | a |
| LA_Bladette de Provence | 12.01 | a |
| FM_Saint Priest le Vernois Rouge | 11.89 | a |
| GS_Chevalier | 11.69 | a |
| LM_Pirénéo | 11.67 | a |
| LA_Renan | 11.59 | a |
| FM_Bladette de Provence | 11.54 | a |
| GS_Bladette de Provence | 11.02 | a |

**2- Ferulic acid content**

**Table S8:** Comparison of the ferulic acid content in bran of durum wheat and bread wheat varieties, harvested in 2017

The results are expressed by the average content in mg/g of dry matter (wheat bran)

G1, G2, G3,G4 express groups of varieties. Varieties with the same letters are not significantly different

| Sample | mean value | G1 | G2 | G3 | G4 |
| --- | --- | --- | --- | --- | --- |
| LA1823 | 1.98 | a |  |  |  |
| Chevalier | 1.79 | a | b |  |  |
| Claudio | 1.58 |  | b | c |  |
| Bidi17 | 1.42 |  |  | c | d |
| Pirénéo | 1.38 |  |  | c | d |
| Rouge de Bordeaux | 1.36 |  |  |  | d |

**Table S9:** Comparison of the ferulic acid content in bran of the bread wheat varieties harvested in 2015, in terroirs GS and LA.

The results are expressed by the average content in mg/g of dry matter (wheat bran)

G1, G2, express groups of varieties. Varieties with the same letters are not significantly different

| Sample | mean value | G1 | G2 |
| --- | --- | --- | --- |
| Chevalier | 2.40 | a |  |
| Bladette de Provence | 2.38 | a |  |
| Renan | 2.13 |  | b |
| Saint Priest le Vernois Rouge | 1.97 |  | b |
| Pirénéo | 1.92 |  | b |
| Redon | 1.91 |  | b |

**Table S10:** Comparison of the ferulic acid content in bran of the bread wheat varieties harvested in 2015, in the fours terroirs GS, LA, LM and FM.

The results are expressed by the average content in mg/g of dry matter (wheat bran)

G1, G2, G3, G4, G5, G6 and G7 express groups of varieties. Varieties with the same letters are not significantly different

| Sample | mean value | G1 | G2 | G3 | G4 | G5 | G6 | G7 |
| --- | --- | --- | --- | --- | --- | --- | --- | --- |
| GS_Bladette de Provence | 2.51 | a |  |  |  |  |  |  |
| GS_Chevalier | 2.43 | a | b |  |  |  |  |  |
| LA_Chevalier | 2.37 | a | b | c |  |  |  |  |
| LA_Bladette de Provence | 2.26 | a | b | c | d |  |  |  |
| FM_Bladette de Provence | 2.18 | a | b | c | d | e |  |  |
| GS_Renan | 2.16 | a | b | c | d | e | f |  |
| GS_Saint Priest le Vernois Rouge | 2.13 |  | b | c | d | e | f |  |
| LA_Renan | 2.11 |  | b | c | d | e | f |  |
| LM_Chevalier | 2.04 |  |  | c | d | e | f | g |
| LM_Renan | 2.04 |  |  | c | d | e | f | g |
| GS_Pirénéo | 2.02 |  |  | c | d | e | f | g |
| LM_Pirénéo | 2.01 |  |  | c | d | e | f | g |
| LA_Redon | 1.96 |  |  |  | d | e | f | g |
| FM_Saint Priest le Vernois Rouge | 1.90 |  |  |  | d | e | f | g |
| GS_Redon | 1.86 |  |  |  |  | e | f | g |
| LA_Pirénéo | 1.81 |  |  |  |  |  | f | g |
| LA_Saint Priest le Vernois Rouge | 1.81 |  |  |  |  |  | f | g |
| FM_Redon | 1.69 |  |  |  |  |  |  | g |

**Table S11:** Comparison of total ferulic acid content in sourdoughs. G1, G2, G3 express groups of differences. Sourdoughs with the same letters are not significantly different

| Sourdough | mean value | G1 | G2 | G3 |
| --- | --- | --- | --- | --- |
| CRA | 0.52 | a |  |  |
| EDI | 0.30 |  | b |  |
| STE | 0.18 |  |  | c |

**Table S12**: Comparison of total ferulic acid content in different leavens of doughs. G1, G2 express groups of differences. Leavens with the same letters are not significantly different

| Leaven type | mean value | G1 | G2 |
| --- | --- | --- | --- |
| S_CRA | 0.28 | a |  |
| Y_INS | 0.26 | a | b |
| S_CRA x Y_HIR | 0.26 | a | b |
| Y_BIO | 0.26 | a | b |
| S_EDI | 0.26 | a | b |
| Y_HIR | 0.25 |  | b |
| S_STE | 0.24 |  | b |
| S_STE x Y_HIR | 0.24 |  | b |

**Table S13**: Comparison of total ferulic acid content in different leaven types of outer bread samples (crumb + crust). G1,G2,G3 express groups of differences. Leavens with the same letters are not significantly different

| Leaven type | mean value | G1 | G2 | G3 |
| --- | --- | --- | --- | --- |
| S_CRA | 0.33 | a |  |  |
| Y_BIO | 0.31 | a | b |  |
| S_EDI | 0.30 | a | b |  |
| Y_HIR | 0.29 |  | b | c |
| S_CRA x Y_HIR | 0.28 | a | b | c |
| S_STE x Y_HIR | 0.28 |  | b | c |
| Y_INS | 0.27 |  | b | c |
| S_STE | 0.26 |  |  | c |

**Table S14**: Comparison of total ferulic acid content in different leaven types of inner bread samples (crumb). G1 express groups of differences. Leavens with the same letters are not significantly different

| Leaven type | mean value | G1 |
| --- | --- | --- |
| S_CRA | 0.25 | a |
| Y_BIO | 0.25 | a |
| S_EDI | 0.24 | a |
| S_CRA x Y_HIR | 0.25 | a |
| Y_HIR | 0.25 | a |
| S_STE | 0.24 | a |
| S_STE x Y_HIR | 0.22 | a |
| Y_INS | 0.22 | a |
